# Identification of potential mitochondrial CLPXP protease interactors and substrates suggests its central role in energy metabolism

**DOI:** 10.1038/srep18375

**Published:** 2015-12-17

**Authors:** Fabian Fischer, Julian D. Langer, Heinz D. Osiewacz

**Affiliations:** 1 Johann Wolfgang Goethe University, Faculty for Biosciences & Cluster of Excellence ‘Macromolecular Complexes’ Frankfurt, Institute for Molecular Biosciences, Max-von-Laue-Str. 9, 60438 Frankfurt, Germany; 2 Department of Molecular Membrane Biology, Max Planck Institute of Biophysics, Max-von-Laue-Str. 3, 60438 Frankfurt, Germany

## Abstract

Maintenance of mitochondria is achieved by several mechanisms, including the regulation of mitochondrial proteostasis. The matrix protease CLPXP, involved in protein quality control, has been implicated in ageing and disease. However, particularly due to the lack of knowledge of CLPXP’s substrate spectrum, only little is known about the pathways and mechanisms controlled by this protease. Here we report the first comprehensive identification of potential mitochondrial CLPXP *in vivo* interaction partners and substrates using a combination of tandem affinity purification and differential proteomics. This analysis reveals that CLPXP in the fungal ageing model *Podospora anserina* is mainly associated with metabolic pathways in mitochondria, e.g. components of the pyruvate dehydrogenase complex and the tricarboxylic acid cycle as well as subunits of electron transport chain complex I. These data suggest a possible function of mitochondrial CLPXP in the control and/or maintenance of energy metabolism. Since bioenergetic alterations are a common feature of neurodegenerative diseases, cancer, and ageing, our data comprise an important resource for specific studies addressing the role of CLPXP in these adverse processes.

Mitochondria are essential eukaryotic organelles involved in different metabolic processes like energy conversion or the synthesis of iron sulfur clusters^1^, in cellular signalling^2^, and the control of apoptosis^3^,^4^. Not surprisingly, owing to their central role in cellular physiology, dysfunction of mitochondria and changes in mitochondrial bioenergetics are a common feature of neurodegenerative diseases^5^, cancer^6^, and ageing^7^,^8^,^9^. A complex network of different quality control pathways is active in an effort to maintain mitochondrial function^10^,^11^ and adapt it to stress conditions^12^. In this network, mitochondrial proteases are increasingly recognized as important regulatory components and no longer viewed as mere degradation machineries for damaged proteins^13^. To fully appreciate the biological function of mitochondrial proteases, in-depth knowledge of their substrates and interaction partners is necessary. One of the least characterized players in this regard is the soluble matrix serine protease CLPXP.

Like its bacterial counterpart, mitochondrial CLPXP is composed of a 14-mer CLPP proteolytic chamber, formed by two heptameric CLPP rings, and one or two hexameric rings of the AAA+ chaperone CLPX which recognizes and unfolds substrate proteins^14^. The proteolytic component CLPP participates in the mitochondrial unfolded protein response (UPR^mt^), a mitochondria-to-nucleus stress signalling pathway. In this context CLPP is thought, mostly based on observations made in *C*. *elegans*, to initiate the UPR^mt^ by degrading misfolded proteins that accumulate under stress conditions^15^. In addition, a recent study applied a proximity-dependent labeling technique in human cells to determine 48 potential CLPP-interacting proteins. Although this particular technique does not allow discrimination of near-neighbors or direct physical interaction partners of the ‘bait’ protein^16^, it is intriguing that most identified proteins were components of the ETC or enzymes involved in mitochondrial metabolism^17^. However, direct interaction partners and, most importantly, potential *in vivo* substrates of the mitochondrial CLPXP protease remain as yet undetermined and its biological role is thus only very superficially understood. In part, this lack of knowledge is due to the fact that the proteolytic component CLPP is absent in *Saccharomyces cerevisiae*^14^, the most widely used eukaryotic model organism to address such foundational research questions.

In the fungal ageing model *Podospora anserina*^18^, deletion of the gene *PaClpP*, encoding the proteolytic subunit of CLPXP, led to an unexpected increase of the mutant strain’s healthy lifespan^19^. Importantly, it was possible to revert this longevity phenotype by expression of human *ClpP* in the fungal deletion background, demonstrating functional conservation of human and fungal CLPP. These features, together with well-established methods for experimental manipulation and genetic, biochemical, and cell biology analysis, make *P*. *anserina* a promising model organism to investigate conserved biological roles of mitochondrial CLPXP proteases.

In the present study, we set out to characterize the potential *in vivo* substrates and interaction partners of a chimeric human CLPP fungal CLPX protease in *P*. *anserina* using an unbiased approach. This was achieved with an experimental strategy originally developed for the identification of bacterial CLP protease substrates^20^ which, to our knowledge, has to date not been applied in eukaryotes. Overall, we uncovered at least 19 potential CLPXP substrates as well as more than 40 potential CLPP interaction partners. The vast majority of these proteins belong to fundamental mitochondrial metabolic pathways. Prominent potential targets of CLPXP that were identified are components of the pyruvate dehydrogenase complex and the tricarboxylic acid cycle, subunits of electron transport chain complex I, and enzymes involved in amino acid and fatty acid metabolism. These data strongly suggest that mitochondrial CLPXP in *P*. *anserina* functions in the control and/or maintenance of mitochondrial energy metabolism, a role that might be conserved across eukaryotic species, including humans.

## Results

### Establishing a CLPP substrate-trapping assay in *P*. *anserina*

The CLPP substrate-trapping assay is a well-established experimental strategy to comprehensively identify CLP protease substrates in an unbiased manner. This combined biochemical and proteomics approach was originally developed in *Escherichia coli*, where it served to reveal more than 100 potential substrates of CLPXP^20^,^21^ and was subsequently applied to successfully characterize the substrate spectra of other bacterial CLP proteases^22^,^23^. Briefly, tagged and catalytically inactive CLPP is used to trap and enrich substrates in the proteolytic chamber *in vivo*, followed by CLPP purification and mass spectrometry-based identification of potential substrates. Essential requirements to implement the assay are a strain deleted for wild-type *ClpP* which harbors mutationally inactivated CLPP plus the retained ability of inactive CLPP to oligomerize. Given these conditions, the CLP protease’s chaperone component is still able to translocate substrates into the CLPP proteolytic chamber but, since CLPP is now catalytically inactive, substrates can no longer be degraded and are thus trapped by CLPP. To date, no attempts to adopt this assay in a eukaryotic system have been reported.

**Figure 1:**
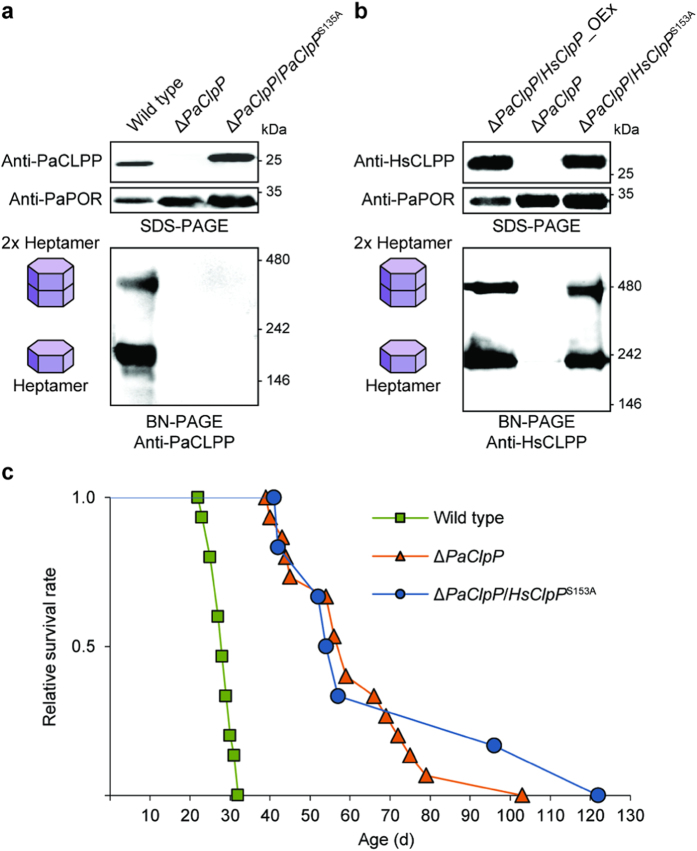
Catalytically inactive human CLPP in *P*. *anserina* is suitable for use in a CLPP substrate-trapping assay. (**a**) Western blot analyses using a *P*. *anserina* CLPP-specific antibody after separation of mitochondrial protein extracts from the indicated strains with SDS-PAGE (above) or BN-PAGE (below). PaPORIN was detected as a loading control. Western blot analysis after SDS-PAGE reveals that monomeric, catalytically inactive PaCLPP (PaCLPP^S135A^) has an increased size compared to the wild-type PaCLPP monomer (predicted size of mature protein: ~25 kDa). After BN-PAGE, two distinct PaCLPP oligomers, corresponding to the heptameric CLPP ring (at around ~170 kDa) and the full proteolytic chamber formed by two heptamers (at around ~340 kDa), are only visible in the wild-type sample. (**b**) Western blot analyses as in a, using a *Homo sapiens* CLPP-specific antibody and mitochondrial protein extracts from the indicated strains. Monomeric, catalytically inactive HsCLPP (HsCLPP^S153A^) has the same size as the wild-type HsCLPP monomer (predicted size of mature protein: ~24 kDa) and, like wild-type HsCLPP, is able to form heptameric rings (at around ~220 kDa) and the full 14-mer proteolytic chamber (at around ~440 kDa) in *P*. *anserina* mitochondria. **c**, Lifespan of wild type (28.2 ± 0.7; n = 15), Δ*PaClpP* (61.3 ± 4.3; n = 15; *P* = 3.4E-06), and Δ*PaClpP*/*HsClpP*^S153A^ (70.5 ± 12.8; n = 6; *P* = 3.7E-05) isolates at 27 °C. Data given in parentheses are mean lifespan ± s.e.m. in days. *P*-values were determined in comparison to the wild-type sample by two-tailed Wilcoxon rank-sum test.

We set out to establish a CLPP substrate-trapping assay using the already available *P*. *anserina ClpP* deletion strain (Δ*PaClpP*)^19^,^24^ to identify potential *in vivo* substrates and interaction partners of mitochondrial CLPXP. In a first attempt, a *P*. *anserina* CLPP variant (PaCLPP^S135A^), inactivated by mutating its catalytic serine to alanine, was introduced into Δ*PaClpP*. Western blot analyses of mitochondrial protein extracts from the resulting strain (Δ*PaClpP*/*PaClpP*^S135A^) revealed that inactive PaCLPP was present in the fungal mitochondria but apparently failed to form the proteolytic chamber necessary for the trapping of substrates *in vivo* (Fig. 1a). Additionally, an increase in the size of monomeric PaCLPP^S135A^ compared to wild-type PaCLPP was observed. The altered size of inactive PaCLPP indicates a possible autocatalytic processing, i.e. possibly the self-cleavage of a propeptide, of this protease, which is blocked upon its catalytic inactivation. Indeed, autocatalytic cleavage of a propeptide has early on been described for *E*. *coli* CLPP^25^. Taken together, these results indicated that PaCLPP was not suitable for use in the CLPP substrate-trapping assay.

We have previously demonstrated that human CLPP can functionally complement for the absence of PaCLPP, as it was able to completely revert Δ*PaClpP*’s longevity phenotype when expressed in the fungal *PaClpP* deletion background (resulting in the strain Δ*PaClpP*/*HsClpP*_Oex)^19^. Thus, use of HsCLPP represented a possible alternative to execute the intended assay in *P*. *anserina*. After introducing inactive human CLPP (HsCLPP^S153A^) into Δ*PaClpP*, the protein was present in mitochondria from the resulting transgenic fungal strain (Δ*PaClpP*/*HsClpP*^S153A^) and appeared identical in size to wild-type HsCLPP in the mitochondria from Δ*PaClpP*/*HsClpP*_Oex (Fig. 1b). This indicates, congruent with earlier observations^26^, that human CLPP might in fact not undergo autocatalytic processing.

Most importantly, HsCLPP^S153A^, like wild-type HsCLPP, was still able to form the full 14-mer proteolytic chamber inside mitochondria from the *PaClpP* deletion strain (Fig. 1b). However, most likely reflecting its catalytic inactivation, inactive HsCLPP was not able to revert the lifespan of Δ*PaClpP* (Fig. 1c). It should be noted that the human CLPP oligomers appeared larger than their theoretically expected size. This phenomenon was already noted in a previous study^19^ and is likely explained by the fact that the migration of proteins during BN-PAGE is significantly influenced by their native shape, their intrinsic charge and the amount of bound Coomassie dye^27^. Taken together, inactive human CLPP appeared to fulfill the necessary prerequisites to be used for a CLPP substrate-trapping assay in *P*. *anserina*.

We also attempted to replace the *P*. *anserina* CLPX chaperone with its human homologue, but human CLPX was not stable in the fungal mitochondria (Supplementary Fig. 1). Therefore, we used a strain which harbors the inactive human CLPP protease and the *P*. *anserina* CLPX chaperone to perform the CLPP substrate-trapping in *P*. *anserina*.

**Figure 2:**
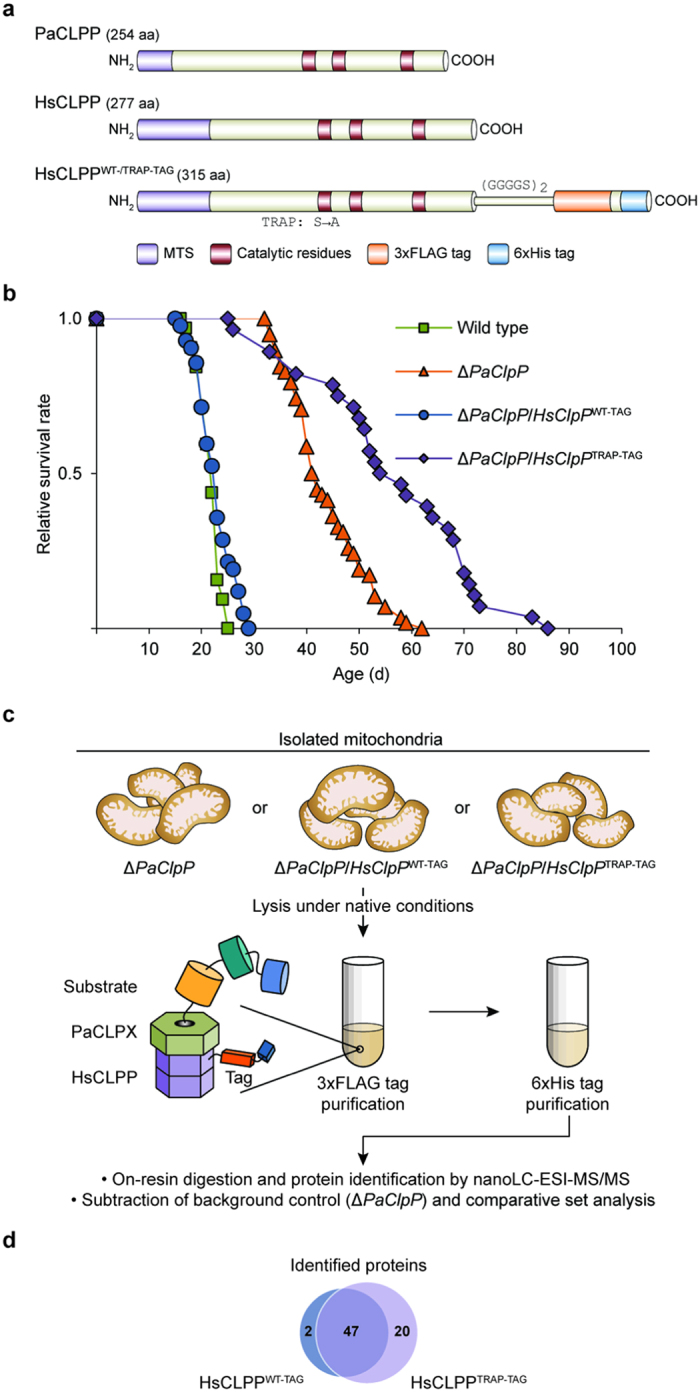
CLPP substrate-trapping assay using isolated mitochondria. (**a**) Cartoon presentation of *P*. *anserina* and human CLPP homologues as well as recombinant HsCLPP variants. PaCLPP is 254 amino acids and HsCLPP 277 amino acids long. Both CLPP homologues display a conserved distribution of the canonical catalytic residues Ser, His, and Asp and contain a N-terminal mitochondrial targeting sequence (MTS). Recombinant human CLPP with a C-terminal (GGGGS)_2_ linker followed by a 3xFLAG-6xHis-tag has a length of 315 amino acids. Catalytic inactivation of recombinant HsCLPP was achieved by mutating its catalytic serine at position 153 of the full-length HsCLPP pre-protein to alanine. (**b**) Lifespan of wild type (21.8 ± 0.4; n = 32), Δ*PaClpP* (43.7 ± 1.0; n = 58; *P* = 8.3E-25), Δ*PaClpP*/*HsClpP*^WT-TAG^ (22.7 ± 0.5; n = 42; *P* = 3.7E-01), and Δ*PaClpP*/*HsClpP*^TRAP-TAG^ (56.9 ± 2.9; n = 28; *P* = 1.9E-17) isolates at 27 °C. Data given in parentheses are mean lifespan ± s.e.m. in days. *P*-values were determined in comparison to the wild-type sample by two-tailed Wilcoxon rank-sum test. (**c**) Overview of CLPP substrate-trapping assay and proteomics work flow. (**d**) Venn diagram displaying overlap of identified proteins co-purifying with 3xFLAG-6xHis-tagged active (HsCLPP^WT-TAG^) or inactive human CLPP (HSCLPP^TRAP-TAG^).

For human CLPP purification, we constructed expression vectors coding for a C-terminally 3xFLAG-6xHis-tagged wild-type (HsCLPP^WT-TAG^) or inactive HsCLPP variant (HsCLPP^TRAP-TAG^; Fig. 2a). Transformation of each vector into Δ*PaClpP* spheroblasts and selection of transformants with single genomic vector integrations yielded the strains Δ*PaClpP*/*HsClpP*^WT-TAG^ and Δ*PaClpP*/*HsClpP*^TRAP-TAG^, respectively (Supplementary Fig. 2). Presence of recombinant affinity-tagged active or inactive HsCLPP in the fungal mitochondria was validated by western blot analyses (Supplementary Fig. 2). Lifespan of Δ*PaClpP*/*HsClpP*^WT-TAG^ was again identical to that of the *P*. *anserina* wild-type strain, directly confirming that the affinity tag doesn’t interfere with HsCLPP’s function inside the fungal mitochondria (Fig. 2b). At this point it should be emphasized that *P*. *anserina* represents a unique system for analyses of eukaryotic CLP proteases *in vivo*, since the deletion of *PaClpP* leads to a very specific phenotype: a pronounced increase in lifespan^19^. This easily detectable phenotype allows direct assessment of the functionality of different CLPP variants and constructs *in vivo*. In contrast to HsCLPP^WT-TAG^, we found that HsCLPP^TRAP-TAG^, as expected due to its catalytic inactivation, was not able to revert the extended lifespan of Δ*PaClpP*.

For execution of the assay, mitochondria isolated from biological triplicates of the strains Δ*PaClpP*, Δ*PaClpP*/*HsClpP*^WT-TAG^, or Δ*PaClpP*/*HsClpP*^TRAP-TAG^ were used for native tandem affinity purification, which included rigorous washing steps to prevent unspecific adsorption to the bed material. Purified proteins were on-resin digested and identified by nanoLC-ESI-MS/MS (Fig. 2c). Stringent filtering criteria ensured that only reproducibly co-purifying proteins, found in at least 2 out of 3 independent biological replicates of the respective sample, were used for subsequent analyses (see Methods section for details). Unspecifically purifying proteins also present in the Δ*PaClpP* background control sample were subtracted from the sets of proteins co-purifying with either HsCLPP^WT-TAG^ or HsCLPP^TRAP-TAG^ (Supplementary Table 1). The remaining proteins that specifically co-purified with both HsCLPP variants over the background control sample were classified as potential interaction partners of CLPP. All proteins that fulfilled the selective criterion of co-purifying either exclusively or highly enriched with the catalytically inactive variant HsCLPP^TRAP-TAG^, as to be expected for proteins trapped by inactive CLPP *in vivo*, were classified as potential CLPXP substrates.

### Mitochondrial CLPXP is predominantly associated with metabolic pathways

Overall, we identified 47 proteins specifically co-purifying with both HsCLPP variants over the background control sample (termed HsCLPP^WT-/TRAP-TAG^-shared; Fig. 2d and Table 1), which were therefore classified as potential interaction partners of CLPP in *P*. *anserina*. Notably, 12 of these 47 shared proteins were enriched >1.5-fold in the Δ*PaClpP*/*HsClpP*^TRAP-TAG^ sample (termed HsCLPP^TRAP-TAG^-enriched; Table 1). Only two proteins, homologues of the human methylcrotonoyl-CoA carboxylase beta chain (MCCC2; Swiss-Prot ID: Q9HCC0) and of a fungal cytochrome c peroxidase (ccp-1; Swiss-Prot ID: Q7SDV9), were preferentially co-purified with HsCLPP^WT-TAG^ (Fig. 2d). Because their abundance was very low and they were also present in one biological replicate of the Δ*PaClpP*/*HsClpP*^TRAP-TAG^ sample, these two proteins were excluded from subsequent analyses.

Most strikingly, 18 proteins were exclusively associated with HsCLPP^TRAP-TAG^ (i.e. they were not co-purified with active HsCLPP^WT-TAG^) and two additional proteins were highly enriched (enrichment factor >3) in the Δ*PaClpP*/*HsClpP*^TRAP-TAG^ sample over the Δ*PaClpP*/*HsClpP*^WT-TAG^ sample. Hence, 20 proteins were identified that co-purified either exclusively or highly enriched with the catalytically inactive variant HsCLPP^TRAP-TAG^ (termed HsCLPP^TRAP-TAG^-specific, Fig. 2d and Table 2). Notably, one of the HsCLPP^TRAP-TAG^-specific proteins was PaCLPX, thus confirming that inactive, affinity-tagged human CLPP, as a necessary prerequisite for the trapping of substrates, retains the ability to interact with the chaperone PaCLPX. A similar association of CLPX only with inactive but not with wild-type CLPP after stringent affinity purification, which most likely reflects stabilization of the CLPP-CLPX interaction in the presence of trapped substrates^28^, was previously observed in a prokaryotic CLPP substrate-trapping assay^22^. The remaining 19 HsCLPP^TRAP-TAG^-specific proteins were classified as potential CLPXP substrates in *P*. *anserina* (Table 2).

**Figure 3:**
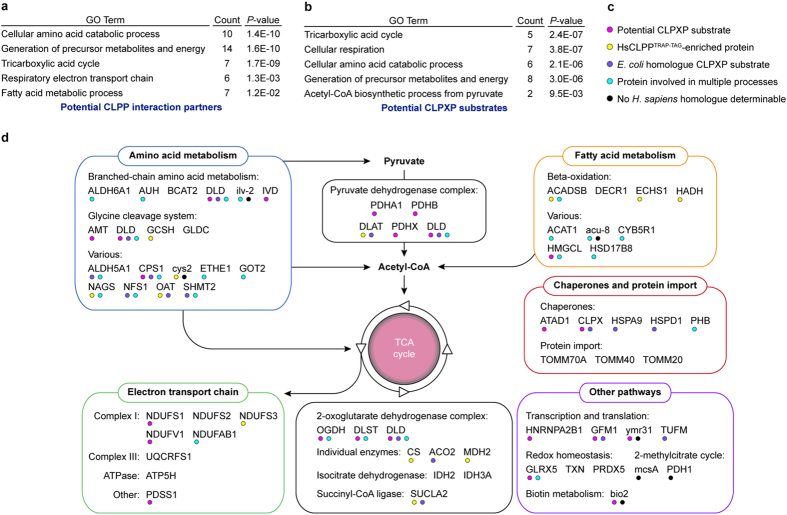
Potential mitochondrial CLPP interaction partners and CLPXP substrates. (**a**) Selected GO terms enriched among human homologues of genes coding for proteins co-purifying with both HsCLPP variants (HsCLPP^WT-/TRAP-TAG^-shared) that were classified as potential CLPP interaction partneres. Count refers to the number of genes associated with the respective GO term. Only GO terms with Bonferroni-corrected *P*-values <5.0E-02 were considered statistically significant. For full GO enrichment analysis data see Supplementary Table 4. (**b**) Selected GO terms enriched among human homologues of genes coding for potential CLPXP substrates. For full GO term enrichment analysis data see Supplementary Table 5. **c**, Legend for colour-coded circles indicating different characteristics of the identified proteins. (**d**) Overview of proteins identified as HsCLPP^WT-/TRAP-TAG^-shared (Table 1), HsCLPP^TRAP-TAG^-enriched (Table 1) or potential CLPXP substrates (Table 2). Proteins are denoted by their corresponding gene names which are listed in Tables 1 and 2. Colour-coded circles below each protein indicate its respective characteristics. For example, the TCA cycle enzyme dihydrolipoyl dehydrogenase (DLD; Swiss-Prot ID: P09622; Table 2) was identified as a potential CLPXP substrate in our analysis (magenta circle), its bacterial homologue is a known *E*. *coli* CLPXP substrate (purple circle), and it is involved in multiple cellular processes (blue circle). Proteins with no associated circles, e.g. the mitochondrial peroxiredoxin-5 (PRDX5; Swiss-Prot ID: P30044; Table 1), were identified as potential CLPP interaction partners (i.e. they were specifically co-purified with both HsCLPP variants) but do not possess any of the additional characteristics.

For the vast majority of identified proteins, human homologues, which were used for data annotation and analyses, were readily determinable (Supplementary Tables 2 and 3). Gene ontology (GO) analysis unveiled marked enrichment of essential mitochondrial metabolic pathways, e.g. the tricarboxylic acid (TCA) cycle and amino acid metabolism, among the genes encoding HsCLPP^WT-/TRAP-TAG^-shared and HsCLPP^TRAP-TAG^-specific proteins (Fig. 3a,b and Supplementary Tables 4 and 5).

Proteins were assigned to their biological process, functional class, and/or associated protein complex (overview in Fig. 3c,d). Several of the proteins identified in our assay were chaperones or belonged to various mitochondrial processes, e.g. control of transcription, translation, and redox homeostasis. Unexpectedly, three components (TOMM20, TOMM40, and TOMM70A) of the translocase of the outer membrane (TOM) complex co-purified with both HsCLPP variants. This possibly indicates that a fraction of purified HsCLPP^WT-TAG^ and HsCLPP^TRAP-TAG^ might in fact be transport intermediates. Altogether 19 proteins were involved in amino acid metabolism, e.g. components of the glycine cleavage system or enzymes involved in the breakdown of branched-chain amino acids. Eight of these were classified as potential CLPXP substrates or were proteins markedly enriched in the Δ*PaClpP*/*HsClpP*^TRAP-TAG^ sample. Likewise, four of the nine proteins related to beta-oxidation or other pathways of fatty acid metabolism were determined as HsCLPP^TRAP-TAG^-specific or HsCLPP^TRAP-TAG^-enriched. Strikingly, nearly all components of the pyruvate dehydrogenase complex (PDC), which is considered a mitochondrial gatekeeper linking glycolysis with mitochondrial energy metabolism, were identified as potential substrates of CLPXP in our analysis. This strongly suggests that the PDC in *P*. *anserina* is one of the major proteolytic targets of mitochondrial CLPXP. The TCA cycle appears to be another prominent target, with many of its enzymes identified as potential CLPXP substrates or potential CLPP-interacting proteins. The TCA cycle lies downstream of amino acid/fatty acid catabolism and the PDC, supplying the reducing equivalents to drive oxidative phosphorylation at the electron transport chain (ETC). Appropriately enough, core subunits of ETC complex I constitute yet another prevalent group containing several of the identified potential CLPXP substrates. Based on the overall results of the CLPP substrate-trapping assay we suggest that CLPXP in *P*. *anserina*, mainly through proteolytic control of PDC, TCA cycle, and ETC components, has a dedicated role in the control and/or maintenance of mitochondrial energy metabolism.

In line with this interpretation of our data, bacterial CLP proteases also degrade numerous metabolic enzymes^20^,^21^,^23^,^29^. In fact, bacterial homologues of 15 proteins we identified, e.g. of the PDC E2 component (DLAT; Swiss-Prot ID: P10515; Table 1) or the TCA cycle enzyme dihydrolipoyl dehydrogenase (DLD; Swiss-Prot ID: P09622; Table 2), are known substrates or interactors of *E*. *coli* CLPXP^20^,^21^. Strikingly, the murine homologue of one of these proteins, the carbamoyl-phosphate synthetase I (CPS1; Swiss-Prot ID: P31327; Tables 1 and 2), also preferentially interacted with a mutated version of human CLPX that lacks ATPase activity, indicating it as a putative physiological substrate of the human chaperone component^30^. As this protein is therefore identified in, including our own, three independent studies as a potential CLPXP-substrate in different organisms, it might in fact represent a substrate of this protease that is highly conserved during evolution. Furthermore, 48 potential human CLPP-interacting proteins were recently identified, most of them components of the ETC or enzymes involved in mitochondrial metabolism^17^. Overall, these observations strongly support a possible biological role of mitochondrial CLPXP connected to the energy metabolism in different eukaryotic organisms, which is already present in its bacterial ancestor.

### The role of mitochondrial CLPXP in human disease

Strikingly, a number of previous studies implicate mitochondrial CLPXP in different human pathologies. We therefore further analysed our results to determine whether they might be of relevance in this regard and could shed light on certain observations concerning CLPP as reported in the literature.

Among the CLPP-associated pathologies is Friedreich’s Ataxia (FRDA), a neurodegenerative disease caused by failed assembly of Fe-S clusters due to defects in the mitochondrial iron chaperone frataxin^31^. In a FRDA mouse model, the proteolytic component CLPP is upregulated at mid-stage of the disease. This upregulation is concomitant with a loss of mitochondrial Fe-S proteins, indicating they are targets of CLPP in FRDA^32^. Indeed, several proteins we identified in our study contain or bind to Fe-S clusters, e.g. aconitase (ACO2; Swiss-Prot ID: Q99798; Table 1), biotin synthase (bio2; Swiss-Prot ID: O59778; Table 2), and complex I components such as the NADH-ubiquinone oxidoreductase 75 kDa subunit (NDUFS1; Swiss-Prot ID: P49821; Table 2). In addition, three of the proteins found as CLPXP interactors or substrates, the cysteine desulfurase NSF1 (Swiss-Prot ID: Q9Y697; Table 1), the chaperone HSPA9 (Swiss-Prot ID: P38646; Table 1), and the glutaredoxin-related protein 5 (GLRX5; Swiss-Prot ID: Q86SX6; Table 2), are known to be essential for Fe-S cluster biogenesis in eukaryotic cells including those of mammals^33^. Thus, our findings support the idea that CLPXP has a functional role in FRDA and might possibly be involved in regulating Fe-S cluster assembly and Fe-S cluster proteins.

In human Perrault syndrome (PS), characterized by sensorineural hearing loss and ovarian failure, *ClpP* mutations are assumed as causative for the disease^34^. *ClpP* knockout mice recapitulate the PS-associated phenotype, have severe growth retardation, and show bioenergetic and respiratory deficits in several tissues^35^. The finding that CLPXP is predominantly associated with metabolic pathways is well in concordance with these observations and suggests that PS could partially be driven by a dysregulation of mitochondrial energy metabolism following functional mutations in *ClpP*.

Finally, CLPP was found altered in different tumor types^36^,^37^,^38^, suggesting a link between mitochondrial CLPXP and cancer^38^. For instance, a recent study reported *ClpP* as overexpressed in 45% of 511 primary acute myeloid leukemia (AML) patient samples^17^. *ClpP*-knockdown in AML cell lines with high *ClpP* expression reduced their viability, coinciding with impaired oxidative phosphorylation^17^. These results, certainly in agreement with the main conclusion of our study, strongly imply a role of mitochondrial CLPXP in the control and/or maintenance of mitochondrial energy metabolism as well. It thus is reasonable to speculate that CLPXP, as a regulator of mitochondrial metabolism, is pathogenically altered in at least a subset of cancers and may in fact be involved in mediating and maintaining tumorigenic metabolic reprogramming.

## Discussion

The CLPXP protease is, with a few exceptions, nearly ubiquitously present in mitochondria of eukaryotes where it participates in protein quality control^13^,^14^. Importantly, as exemplified above, the proteolytic component CLPP was found altered in different human diseases, making CLPXP an important target for future research. The precise biological role of CLPXP and thus the underlying molecular mechanisms of CLPP-associated pathologies, however, are barely understood. So far, the only well-elaborated pathway known to involve CLPP is the mitochondrial unfolded protein response in *C*. *elegans*, where CLPP degrades misfolded proteins during upstream UPR^mt^ signalling^15^. Unspecific degradation of damaged proteins as well as processing of protein precursors were long thought to be the main functions of mitochondrial proteases. However, it is rapidly becoming clear that most proteases also perform highly regulated proteolytic activities, thereby influencing mitochondrial function, integrity, and homeostasis^13^.

**Figure 4:**
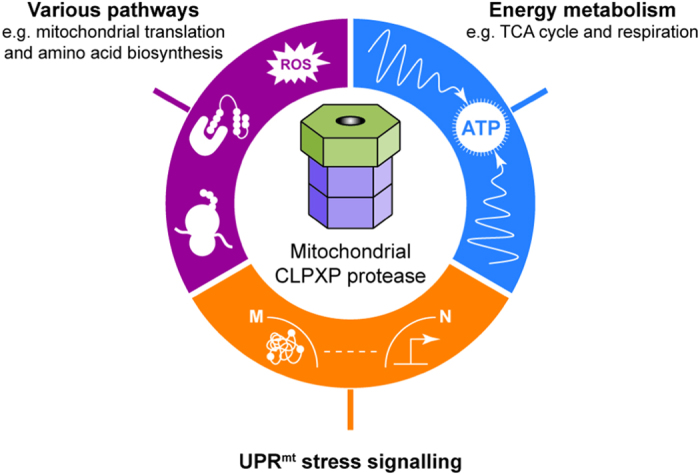
The different roles of mitochondrial CLPXP. The proteolytic component CLPP degrades misfolded mitochondrial proteins in *C*. *elegans* to initiate UPR^mt^ stress signalling, which leads to the induction of nuclear-encoded mitochondrial protective genes^15^. Our study identifies specific proteins and pathways that are likely targets of CLPXP, suggesting a dedicated role of this protease in regulation and maintenance of mitochondrial energy metabolism. The observation that CLPP-knockdown in cancer cells impairs oxidative phosphorylation is in support of this emerging view^17^. Furthermore, several chaperones and components of other mitochondrial pathways, e.g. those controlling translation, redox homeostasis, and amino acid biosynthesis, were found as additional potential targets or interactors of CLPXP.

Overall, our as yet unprecedented initial identification of mitochondrial CLPXP’s potential *in vivo* interaction partners and substrates contributes to a more detailed view of this as yet ill-defined protease. Notwithstanding the necessity of the independent validation of selected potential interaction partners and substrates in future studies, our results clearly argue for a function of *P*. *anserina* CLPXP in specifically targeting most of the central mitochondrial metabolic pathways (Fig. 4). This proposed novel role is in good agreement with correlative evidence in the literature and can be expected to be at least partially conserved during eukaryotic evolution.

Seemingly in opposition to this assumption is the fact that deletion or mutation of *ClpP* in mice^35^ or humans^34^ is associated with negative effects, while the *P*. *anserina ClpP* deletion strain in contrast shows an increase in its healthy lifespan^19^. These different outcomes may be due to distinctions in lifestyle and physiology of these organisms, which pose different challenges in adapting to internal or external insult to cellular and organismal homeostasis. It can be speculated, that in all these organisms absence or partial ablation of the mitochondrial CLPXP protease dysregulates mitochondrial and thus also cellular energy metabolism. While more complex eukaryotic organisms would be unable to cope with this adverse effect, as they require a fine-tuned adaptation of their metabolism in different tissues, the impaired mitochondrial integrity and cellular energy homeostasis in *P*. *anserina* could, at least under controlled laboratory conditions, potentially be compensated by activation of other quality control pathways, e.g. autophagy and/or mitophagy. Autophagy in particular is known to be induced in response to disturbances of cellular bioenergetics^39^,^40^ and was demonstrated to be a longevity assurance mechanism in *P*. *anserina*, mediating lifespan-extension under nutrient starvation conditions^41^.

Since dysfunction of mitochondria is associated with degenerative processes across a wide range of organisms, we anticipate the insights described herein to serve as a valuable precedent for detailed studies in various eukaryotic systems addressing CLPXP’s role in quality control and regulated proteolysis of mitochondrial proteins and in particular its impact on ageing and the development of disease.

## Methods

### *P*. *anserina* strains and cultivation

In the present study, the *P*. *anserina* wild-type strain ‘s’^42^, a *P*. *anserina ClpP* deletion strain (Δ*PaClpP*)^24^, a *PaClpP* deletion strain expressing human *ClpP* (Δ*PaClpP*/*HsClpP*_OEx)^19^, newly generated *PaClpP* deletion strains expressing constructs coding for inactive variants of PaCLPP (Δ*PaClpP*/*PaClpP*^S135A^) or HsCLPP (Δ*PaClpP*/*HsClpP*^S153A^), newly generated *PaClpP* deletion strains expressing constructs coding for C-terminally 3xFLAG-6xHis-tagged variants of active HsCLPP (Δ*PaClpP*/*HsClpP*^WT-TAG^) or inactive HsCLPP (Δ*PaClpP*/*HsClpP*^TRAP-TAG^) as well as a newly generated *P*. *anserina ClpX* deletion strain (Δ*PaClpX*) and a newly generated *PaClpX* deletion strain containing a human *ClpX* expression construct (Δ*PaClpX*/*HsClpX*) were used. All mutant strains are in the genetic background of the wild-type strain ‘s’. Strains were grown on standard cornmeal agar (BMM) at 27 °C under constant light^42^, unless otherwise stated.

### Cloning procedures and generation of *P*. *anserina* mutants

To generate a *PaClpP* deletion strain expressing a construct coding for inactive PaCLPP, Δ*PaClpP* spheroblasts were transformed with the plasmid pPaClpPEx^S135A^. This plasmid contains a hygromycin B resistance gene in the pKO7^43^ vector backbone and the full length *PaClpP* gene under control of its wild-type promoter and terminator, with the codon for catalytic serine-135 (TCT) replaced with a codon for alanine (GCC). Transformants were selected for hygromycin B resistance and verified by Southern blot analysis. Strains with a single integration of pPaClpPEx^S135A^ were termed Δ*PaClpP*/*PaClpP*^S135A^. To construct pPaClpPEx^S135A^, the *PaClpP* promoter (~1 kbp of the gene’s upstream region), gene, and terminator (~500 bp of the gene’s downstream region) were amplified from genomic wild-type DNA by PCR using the oligonucleotides ClpPEx5′HindIIIfor (5′-GTAAGCTTTGTGTATCGCCCC GTCCC-3′) and ClpPEx3′KpnIrev (5′-CCGGTACCTTTCATTGGTCCAGCAGC-3′), introducing HindIII and KpnI restriction sites (underlined). The amplicon was cloned into the pKO7 vector backbone (HindIII/KpnI digested) to obtain the plasmid pPaClpPEx2. Subsequently, a codon substitution (catalytic serine-135 to alanine-135) was introduced by PCR amplification of two fragments using the oligonucleotides ClpPEx5′HindIIIfor (see above) and StoA-PaClpP_rev (5′-GGCTGCGGCGCCACCAACGCAC-3′) or StoA-PaClpP_for (5′-ATGGCGGCTATTCTCCTG-3′) and ClpPEx3′KpnIrev (see above) with pPaClpPEx2 as a template. These fragments were cloned into the pKO7 vector backbone (HindIII/KpnI digested), resulting in the plasmid pPaClpPEx^S135A^.

To obtain a constitutive expression vector coding for inactive HsCLPP (catalytic serine-153 to alanine-153), two fragments were amplified by PCR using the oligonucleotides BamHI-HsClpP_3for (5′-TAGGATCCATGTGGCCCGGAATATTG-3′) and S153A-HsClpP_6rev (5′-GGCGGCGGCCTGGCCCACGCA-3′) or S153A-HsClpP_7for (5′-ATGGGCTCCCTGCTTCTC-3′) and XbaI-HsClpP_4rev (5′-ATTCTAGATCAGGTGCTAGCTGGGAC-3′) with pHsClpPEx1^19^ as a template. These fragments were cloned into the pExMtterhph^24^ vector backbone (BamHI/XbaI digested), resulting in the plasmid pHsClpPEx^S153A^. After transformation of Δ*PaClpP* spheroblasts with pHsClpPEx^S153A^, selection and verification of transformants was performed as described above and strains with a single integration were termed Δ*PaClpP*/*HsClpP*^S153A^.

A constitutive expression vector coding for active HsCLPP with a C-terminal (GGGGS)_2_ linker followed by a 3xFLAG-6xHis-tag (full amino acid sequence: GGGGSGGGGS DYKDHDGDYKDHDIDYKDDDDK HHHHHH) was constructed by PCR amplification of two fragments using the oligonucleotides HsClpP-NotI-for (5′-CAGCGGCCGCCGCAGCGG-3′) and HsClpP-L3F6H-rev (5′-CTTGTAGTCGCCGTCGTGGTCCTTGTAGTCGCTGCCGCCGCCGCCGCTGCCGCCGCCGCCGGTGCTAGCTGGGACAGG-3′) or HsClpP-L3F6H-for (5′-GACCACGACATCGACTACAAGGACGACGACGACAAGCACCACCACCACCACCACTAATCTAGAGTTATTCCCTCACTCAC-3′)and TPamT1-HindIII-rev (5′-CGCCAAGCTTACATTTGATCC-3′) with pHsClpPEx1 as template. Subsequent cloning of these fragments into the pHsClpPEx1 vector backbone (NotI/HindIII digested) yielded the plasmid pHsClpP^WT-TAG^. Using pHsClpP^WT-TAG^ as a template, two fragments were amplified by PCR using the oligonucleotides BamHI-HsClpP_3for (see above) and S153A-HsClpP_6rev (see above) or S153A-HsClpP_7for (see above) and TPaMT1-rev (5′-ACCCTAACCGACTAACAGAC-3′). Cloning of these fragments into the pExMtterhph vector backbone (BamHI/XbaI digested) resulted in the plasmid pHsClpP^TRAP-TAG^, coding for inactive HsCLPP (catalytic serine-153 to alanine-153) with a C-terminal (GGGGS)_2_ linker followed by a 3xFLAG-6xHis-tag. After transformation of Δ*PaClpP* spheroblasts with pHsClpP^WT-TAG^ or pHsClpP^TRAP-TAG^, selection and verification of transformants was performed as described above and strains with a single integration of the respective vector were termed Δ*PaClpP*/*HsClpP*^WT-TAG^ or Δ*PaClpP*/*HsClpP*^TRAP-TAG^.

The generation of a *PaClpX* deletion strain was performed using a two-step protocol previously described in detail^44^, using the oligonucleotides KoClpX1 (5′-ATGGTACCTCATCCAGGTGCTCTAAC-3′), KoClpX2 (5′-GGAAGCTTGGTATGCTTGAGCTACTG-3′), KoClpX3 (5′-GGGGCACTAGTAAGGCGTTTTGGGTATGG-3′) and KoClpX4 (5′-AAGCGGCCGCCTCACTGGCAGAATTACG-3′) for PCR amplification of the required 5′- and 3′-flanking regions of the *PaClpX* gene to ultimately obtain a Δ*PaClpX* cosmid. This gene-specific replacement cosmid was used to transform *P*. *anserina* wild-type spheroplasts. Transformants were selected for phleomycin resistance and successful deletion of *PaClpX* was verified by Southern blot analysis. Strains lacking the *PaClpX* gene and instead harbouring the phleomycin resistance gene (*ble*) were termed Δ*PaClpX*. The human *ClpX* cDNA open reading frame was amplified by PCR using the oligonucleotides BamHI-HsClpX-for (5′-TATAATAAGGATCCATGCCCAGCTGCGGTG-3′) and XbaI-HsClpX-rev (5′-CGCGTCTAGATTAGCTGTTTGCAGCATC-3′), introducing BamHI and XbaI restriction sites (underlined). The amplicon was cloned into the pExMtterhph vector backbone (BamHI/XbaI digested). The resulting plasmid pHsClpXEx1 was subsequently transformed into *P. anserina* Δ*PaClpX* spheroplasts. Transformants were selected for hygromycin B resistance and verified by Southern blot analysis. Strains containing a single integration of pHsClpXEx1 were termed Δ*PaClpP*/*HsClpX*.

### Transformation of *P*. *anserina* spheroblasts

Production and regeneration of *P*. *anserina* spheroblasts were performed as previously described^42^,^45^. After integrative transformation of spheroblasts^45^, transformants were selected by growth on regeneration agar containing either 75 μg ml^−1^ hygromycin b or 6 μg ml^−1^ phleomycin.

### Lifespan determination

Experiments to determine the lifespan of *P*. *anserina* strains at 27 °C under constant light on M2 agar race tubes were performed as previously described^42^. The period of linear growth was recorded as lifespan in days.

### Southern blot analysis

Isolation of *P*. *anserina* total DNA, DNA digestion, gel electrophoresis and Southern blotting were performed according to standard protocols. For Southern blot hybridization and detection, Digoxigenin-labelled hybridization probes (DIG DNA Labelling and Detection Kit, Roche Applied Science) were used according to the manufacturer’s protocol.

The *HsClpP*-specific hybridization probe was amplified by PCR using the oligonucleotides HsClpP_1for (5′-ATGTGGCCCGGAATATTG-3′) and HsClpP_2rev (5′-GCGAGTAGATGTCATAGG-3′) and corresponds to nucleotides 124–355 of the *HsClpP* cDNA sequence (GenBank: BC002956.1). The *HsClpX*-specific hybridization probe was amplified by PCR using the oligonucleotides HsClpX_1for (5′-CTGCAAAGAGCTCCTCTTAG-3′) and HsClpX_2rev (5′-ACGGGTGGATGATACAAAGG-3′) and corresponds to nucleotides 208–432 of the *HsClpX* cDNA sequence (GenBank: BC130373.1). The *PaClpX*-specific hybridization probe was amplified by PCR using the oligonucleotides PaClpX_1for (5′-TTTGAGCGCACAGGTTAC-3′) and PaClpX_2rev (5′-CGTCGAGGACAATGATTC-3′) and corresponds to a 530 bp fragment of the *PaClpX* genomic sequence (GenBank: FO904941.1; Region from 993541 to 995395). As a hybridization probe specific for the phleomycin resistance gene (*ble*), the 1293 bp BamHI-fragment of plasmid pKO3^44^ was used.

### Western blot analysis

Mitochondrial protein extracts from *P*. *anserina* strains were isolated according to a previously developed procedure^42^ and further purified by discontinuous sucrose gradient (20-36-50%) ultracentrifugation.

Separation of proteins by SDS-PAGE and subsequent transfer of proteins to PVDF membranes (Immobilon-FL, Merck Millipore) were performed following standard protocols. Blocking and antibody incubation of blotted PVDF membranes were performed according to the Odyssey ‘Western Blot Analysis’ handbook (LI-COR).

Primary antibodies were raised against a PaCLPP (UniProt: B2B591) specific synthetic peptide ([Ac]-CGTMLSADAKEGKH-[OH]; NEP) corresponding to amino acids 242–254 (antibody dilution: 1:400) and the PaPORIN (UniProt: B2B736) full-length protein (NEP; antibody dilution: 1:5,000). The human CLPP antibody (Abcam, product code: ab56455) was raised against an HsCLPP (Swiss-Prot: Q16740) recombinant fragment corresponding to amino acids 178–278 (antibody concentration: 1 μg ml^−1^). The human CLPX antibody (Abcam, product code: ab122644) was raised against an HsCLPX (Swiss-Prot: O76031) recombinant fragment corresponding to amino acids 39–132 (antibody dilution: 1:250). Recombinant active or inactive human CLPP with a C-terminal 3xFLAG-6xHis-tag was detected using a 3xFLAG- (Sigma-Aldrich; product code: F1804; antibody concentration: 1 μg ml^−1^) or a 6xHis-tag antibody (Abcam, product code: ab9136; antibody dilution: 1:1,000).

In all analyses, secondary antibodies conjugated with the infrared dyes IRDye 800CW or IRDye 680CW (LI-COR) were used (antibody dilution: 1:15,000–20,000). The Odyssey Infrared Imaging System (LI-COR) was used for detection of western blots.

### Blue native PAGE

BN-PAGE was performed according to the protocol developed by Schägger and colleagues^46^. Sample preparation and western blotting of BN-PAGE gels was performed as previously described^19^.

### Tandem affinity purification

Mitochondrial protein extracts from three independent biological replicates of the *P*. *anserina* strains Δ*PaClpP*, Δ*PaClpP*/*HsClpP*^WT-TAG^ or Δ*PaClpP*/*HsClpP*^TRAP-TAG^ were obtained as described above. For each individual purification experiment, 1 mg of freshly isolated mitochondria were pelleted by centrifugation at 20,000 g for 10 min at 4 °C in a total volume of 0.5 ml ice-cold mitochondria isotonic buffer (20 mM HEPES-KOH, pH 7.4, 0.6 M sorbitol). After discarding the supernatant, the mitochondrial pellets were flash-frozen in liquid nitrogen and stored at −80 °C until further use.

Mitochondria were lysed at 5 mg ml^−1^ in a total volume of 200 μl FLAG lysis buffer (20 mM HEPES-KOH, pH 7.4, 300 mM KCl, 1 mM EDTA, 10% w/v glycerol, 1x protease inhibitor cocktail set IV from Calbiochem) with 1% w/v digitonin for 30 min on ice. Lysates were cleared by centrifugation at 20,000 g for 10 min at 4 °C, adjusted to 1 ml with lysis buffer and incubated with 30 μl packed gel volume of anti-FLAG M2 magnetic beads (Sigma-Aldrich) for 2 hours at 4 °C with rotation. After binding, beads were washed with 3 × 200 μl FLAG wash buffer (20 mM HEPES-KOH, pH 7.4, 300 mM KCl, 10% w/v glycerol, 0.01% w/v digitonin) and elution was performed with 2× 150 μl FLAG elution buffer (20 mM HEPES-KOH, pH 7.4, 300 mM KCl, 10% w/v glycerol) containing 3xFLAG peptide (Sigma-Aldrich) at 150 ng µl^-1^. Pooled eluates were supplemented with imidazole (final concentration: 20 mM) and TWEEN 20 (final concentration: 0.01%) and adjusted to a final volume of 700 μl with FLAG elution buffer without 3xFLAG peptide.

The second round of purification was performed by incubating the adjusted first round eluates with 50 μl ‘Dynabeads for His-tag Isolation and Pulldown’ (Life Technologies/Thermo Fisher Scientific) for 30 min at 4 °C with rotation. After binding, beads were washed with 4 × 300 μl His wash buffer (20 mM HEPES-KOH, pH 7.4, 300 mM KCl, 20 mM imidazole, 10% glycerol, 0.01% TWEEN 20) and transferred to a new reaction tube during the last washing step for subsequent mass spectrometric analysis.

### Peptide mass fingerprinting (PMF)

Dynabeads with bound affinity-purified proteins were washed three times using ABC buffer (50 mM NH_4_HCO_3_, pH 8.5, 300 mM KCl and 10% w/v glycerol). Proteins were subsequently denatured using 6 M urea for 2 min at 70 °C, reduced using 5 mM TCEP (Sigma-Aldrich) for 30 min at room temperature and alkylated using 20 mM iodoacetamide (Thermo Fisher Scientific) again for 30 min at room temperature. The samples were then incubated with the protease Lys-C (final concentration: 4 μg ml^−1^; Roche Pharmaceuticals) overnight at 37 °C. After 1:1 dilution and addition of CaCl_2_ to a final concentration of 1 mM, the samples were incubated with trypsin (final concentration: 10 μg ml^−1^; Serva) at 37 °C overnight. Subsequently, samples were centrifuged at 1,000 g for 5 min at room temperature, acidified using 1% formic acid and purified using C18-ZipTips (Merck Millipore) according to the manufacturer’s protocol. The elution fractions were dried using a SpeedVac (Thermo Fisher Scientfic) and stored at −20 °C until further use.

Proteolytic digests were dissolved in 5% acetonitrile with 0.1% formic acid and loaded on reverse phase columns (trapping column: particle size 3 μm, C18, L = 20 mm; analytical column: particle size <2 μm, C18, L = 50 cm; PepMap, Dionex/Thermo Fisher Scientific) using a nano-HPLC (Dionex – UltiMate 3000 RSLCnano, Thermo Fisher Scientific). Peptides were eluted in gradients of water (buffer A: water with 5% v/v acetonitrile and 0.1% formic acid) and acetonitrile (buffer B: 20% v/v water and 80% v/v acetonitrile and 0.1% formic acid). All LC-MS-grade solvents were purchased from Fluka. Typically, gradients were ramped from 5% to 70% buffer B in 200 minutes at flowrates of 300 nl min^−1^. Peptides eluting from the column were ionised online using a ‘nanoFlex ESI’-source and analysed either in a ‘Orbitrap Elite’ mass spectrometer or in a ‘Q Exactive Plus’ mass spectrometer (Thermo Fisher Scientific). Mass spectra were acquired over the mass range 250–1650 m z^−1^ and sequence information was acquired by computer-controlled, data-dependent automated switching to MS/MS mode using collision energies based on mass and charge state of the candidate ions. All samples were run in technical triplicates.

The data sets were processed using a standard proteomics script with the software ‘Proteome Discoverer 1.4’ (Thermo Fisher Scientific) and exported as mascot generic files. Proteins were identified by matching the derived mass lists against the ‘*P*. *anserina* Genome Project’ database release version 6.32 (downloaded from http://podospora.igmors.u-psud.fr/) on a local Mascot server (Matrix Science, UK). In general, a mass tolerance of 2 ppm for parent ions and 0.5 Da for fragment spectra, two missed cleavages, oxidation of Met, and fixed modification of carbamidomethyl cysteine were selected as matching parameters in the search program. For protein identification using spectral counting, a set of stringent selection criteria were applied to the dataset.

We set a manual Mascot score threshold of 40 and only considered proteins present in at least two of the three independent biological replicates of each sample. Proteins also found in the background control sample (Δ*PaClpP*) were subtracted from the sets of proteins co-purifying with either HsCLPP variant. Manual inspection of the data showed that a small subset of proteins found in all three samples was highly enriched in the Δ*PaClpP*/*HsClpP*^WT-TAG^ and/or the Δ*PaClpP*/*HsClpP*^TRAP-TAG^ sample over the background control sample (using spectral counting, enrichment factor >3) and these proteins were also retained for subsequent analyses. The resulting sets of proteins co-purifying with each of the two HsCLPP variants over the background control sample were compared and proteins classified as specifically co-purifying with HsCLPP^WT-TAG^, HsCLPP^TRAP-TAG^, or with both. Proteins that were highly enriched in the Δ*PaClpP*/*HsClpP*^TRAP-TAG^ sample over the Δ*PaClpP*/*HsClpP*^WT-TAG^ sample (using spectral counting, enrichment factor >3) were also classified as specifically co-purifying with HsCLPP^TRAP-TAG^. The resulting sets were further refined by eliminating all proteins not present with at least more than one unique peptide on average across all biological replicates of the respective sample. The mass spectrometry proteomics data have been deposited to the ProteomeXchange Consortium via the PRIDE partner repository with the dataset identifier PXD003264.

## Additional Information

**Data availability**: Access to the proteome data associated with this manuscript is available upon request.

**How to cite this article**: Fischer, F. *et al.* Identification of potential mitochondrial CLPXP protease interactors and substrates suggests its central role in energy metabolism. *Sci. Rep.* **5**, 18375; doi: 10.1038/srep18375 (2015).

## Supplementary Material

Supplementary Information

## Figures and Tables

**Table 1:** Proteins co-purifying with HsCLPP^WT-TAG^ and HsCLPP^TRAP-TAG^.

*P*. *anserina* ID*	Avg. Number of Unique Peptides HsCLPP^WT-TAG^	Avg. Number of Unique Peptides HsCLPP^TRAP-TAG^	Swiss-Prot ID	Gene	Protein
Chaperones and Protein Import					
Pa_6_2570	27.0	28.3	P38646	HSPA9	Stress-70 protein‡
Pa_6_5750	16.7	13.7	P10809	HSPD1	60 kDa heat shock protein‡
Pa_2_9700	14.3	15.3	O94826	TOMM70A	Mitochondrial import receptor subunit TOM70
Pa_2_10580	9.0	9.3	O96008	TOMM40	Mitochondrial import receptor subunit TOM40
Pa_6_1920	3.0	2.7	Q15388	TOMM20	Mitochondrial import receptor subunit TOM20
Pa_2_12760	1.7	1.3	P35232	PHB	Prohibitin
Metabolism					
Pa_1_22300	12.0	15.3	P23378	GLDC	Glycine cleavage system P protein
Pa_3_10790	11.3	13.0	O95571	ETHE1	Persulfide dioxygenase ETHE1
Pa_5_5970	10.0	9.0	Q99798	ACO2	Aconitate hydratase‡,§
Pa_3_11290	8.7	9.3	P00505	GOT2	Aspartate aminotransferase
Pa_6_1590	8.0	7.0	P24752	ACAT1	Acetyl-CoA acetyltransferase
Pa_3_6780	7.3	14.0	O75390	CS	**Citrate synthase**
Pa_6_2730	7.3	6.3	P50213	IDH3A	Isocitrate dehydrogenase [NAD] subunit alpha
Pa_3_2310	6.7	12.0	P10515	DLAT	**Pyruvate dehydrogenase E2 component**‡
Pa_5_11920	5.7	5.0	Q13825	AUH	Methylglutaconyl-CoA hydratase
Pa_2_1050	5.7	4.3	P78827†	ilv-2	Ketol-acid reductoisomerase
Pa_6_10000	5.0	3.7	C7C436†	mcsA	2-methylcitrate synthase
Pa_1_13140	4.7	6.3	P31327	CPS1	Carbamoyl-phosphate synthetase I‡ (Pa_1_13140 matches to large chain/C-terminal region)
Pa_3_7700	4.3	7.7	Q9P2R7	SUCLA2	**Succinyl-CoA ligase subunit beta**‡
Pa_1_3450	4.0	6.0	Q92506	HSD17B8	Estradiol 17-beta-dehydrogenase 8
Pa_1_17280	3.3	5.0	Q12428†	PDH1	Probable 2-methylcitrate dehydratase
Pa_1_14630	3.0	6.3	Q8N159	NAGS	**N-acetylglutamate synthase**
Pa_4_7010	3.0	3.7	Q16698	DECR1	2.4-dienoyl-CoA reductase
Pa_3_10910	3.0	3.3	P15937†	acu-8	Acetyl-CoA hydrolase
Pa_4_8600	3.0	3.0	O15382	BCAT2	Branched-chain-amino-acid aminotransferase
Pa_2_6200	2.3	5.0	P23434	GCSH	**Glycine cleavage system H protein**
Pa_6_8420	2.3	4.0	P45954	ACADSB	**Short/branched chain specific acyl-CoA dehydrogenase**
Pa_2_430	2.3	3.3	Q9Y697	NFS1	Cysteine desulfurase‡
Pa_3_2600	2.0	5.3	P40926	MDH2	**Malate dehydrogenase**
Pa_1_15690	2.0	3.7	Q10341†	cys2	**Probable serine-O-acetyltransferase cys2**
Pa_2_4980	2.0	3.3	Q16836	HADH	**Hydroxyacyl-coenzyme A dehydrogenase**
Pa_3_1420	2.0	2.0	Q9UHQ9	CYB5R1	NADH-cytochrome b5 reductase 1
Pa_3_9430	1.7	3.0	P04181	OAT	**Ornithine aminotransferase**‡
Pa_4_3040	1.7	1.7	P48735	IDH2	Isocitrate dehydrogenase [NADP]
Pa_1_7660	1.7	1.3	Q02252	ALDH6A1	Methylmalonate-semialdehyde dehydrogenase
Pa_7_10210	1.3	3.0	P30084	ECHS1	**Enoyl-CoA hydratase**
Pa_1_1980	1.3	1.7	P51649	ALDH5A1	Succinate-semialdehyde dehydrogenase‡
Pa_4_660	1.3	1.3	P34897	SHMT2	Serine hydroxymethyltransferase‡
Electron Transport Chain and Respiration					
Pa_1_14370	2.7	2.3	O75947	ATP5H	ATP synthase subunit d
Pa_4_7160	2.0	4.7	O75489	NDUFS3	**NADH dehydrogenase iron-sulfur protein 3**
Pa_1_8620	2.0	1.7	O75306	NDUFS2	NADH dehydrogenase iron-sulfur protein 2§
Pa_5_7500	1.3	1.7	O14561	NDUFAB1	Acyl carrier protein
Pa_6_240	1.3	1.3	P47985	UQCRFS1	Cytochrome b-c1 complex subunit Rieske§
Other Pathways					
Pa_2_12010	8.7	10.0	P49411	TUFM	Mitochondrial elongation factor Tu‡
Pa_4_1130	2.3	2.3	nhd	—	—
Pa_5_8240	2.0	2.7	P30044	PRDX5	Peroxiredoxin-5
Pa_6_8740	2.0	2.3	P10599	TXN	Thioredoxin

^*^P. *anserina* IDs correspond to the ‘*P*. *anserina* Genome Project’ database release version 6.32 (downloaded from http://podospora.igmors.u-psud.fr/).

^†^If no human homologue was determinable, if possible a homologue from a fungal species was selected for reference.

^‡^Protein whose prokaryotic homologue was identified as a substrate of *E*. *coli* CLPXP^20^,^21^.

^§^Fe-S containing/binding protein.

Listed are all proteins that were specifically co-purified with both catalytically active (HsCLPP^WT-TAG^) and inactive human CLPP (HsCLPP^TRAP-TAG^) over the background control sample and therefore classified as potential CLPP interaction partners. For each protein the *P*. *anserina* ID, average number of unique peptides identified by MS analyses across all biological replicates of the respective sample as well as Swiss-Prot ID, and gene and protein name of the human homologue (Supplementary Table 1) are listed. Categories (e.g. ‘Metabolism’) were assigned based on annotations from the Swiss-Prot database and the general literature. nhd, no homologue determinable. Proteins enriched >1.5-fold in the Δ*PaClpP*/*HsClpP*^TRAP-TAG^ sample over the Δ*PaClpP*/*HsClpP*^WT-TAG^ sample (HsCLPP^TRAP-TAG^-enriched) are in bold.

**Table 2:** Proteins identified as potential CLPXP substrates.

*P*. *anserina* ID*	Avg. Number of Unique Peptides†	Swiss-Prot ID	Gene	Protein
Chaperones				
Pa_6_5510	7.3	Q8NBU5	ATAD1	ATPase family AAA domain-containing protein 1
Pa_6_5590	3.7	O76031	CLPX	ATP-dependent Clp protease ATP-binding subunit clpX-like§
Metabolism				
Pa_6_5560	15.7	Q02218	OGDH	2-oxoglutarate dehydrogenase E1 component
Pa_6_1640	7.7	P31327	CPS1	Carbamoyl-phosphate synthetase I§ (Pa_6_1640 matches to small chain/N-terminal region)
Pa_7_9520	7.3	O00330	PDHX	Pyruvate dehydrogenase protein X component
Pa_5_5370	5.0 (x3.8)	P36957	DLST	2-oxoglutarate dehydrogenase E2 component
Pa_7_10050	4.0	P08559	PDHA1	Pyruvate dehydrogenase E1 component subunit alpha
Pa_1_13750	2.7	P48728	AMT	Aminomethyltransferase
Pa_5_5810	2.3	P09622	DLD	Dihydrolipoyl dehydrogenase§
Pa_1_15800	2.0	P11177	PDHB	Pyruvate dehydrogenase E1 component subunit beta
Pa_1_20100	1,3	P26440	IVD	Isovaleryl-CoA dehydrogenase
Pa_3_9520	1.3	P35914	HMGCL	Hydroxymethylglutaryl-CoA lyase
Electron Transport Chain				
Pa_3_4870	21.0	P28331	NDUFS1	NADH dehydrogenase 75 kDa subunit‖
Pa_4_7950	3.7	P49821	NDUFV1	NADH dehydrogenase flavoprotein 1‖
Pa_5_9670	3.3	Q5T2R2	PDSS1	Decaprenyl-diphosphate synthase subunit 1
Other Pathways				
Pa_3_11170	10.7 (x4.0)	O59778‡	bio2	Biotin synthase‖
Pa_1_18430	2.7	P22626	HNRNPA2B1	Heterogeneous nuclear ribonucleoproteins A2/B1
Pa_2_10680	2.0	Q86SX6	GLRX5	Glutaredoxin-related protein 5‖
Pa_1_6330	1.7	Q96RP9	GFM1	Mitochondrial elongation factor G§
Pa_5_2590	1.7	G2TRP3‡	ymr31	Mitochondrial 37S ribosomal protein YMR-31

^*^P. *anserina* IDs correspond to the ‘*P*. *anserina* Genome Project’ database release version 6.32 (downloaded from http://podospora.igmors.u-psud.fr/).

^†^If the protein was also co-purified with HsCLPP^WT-TAG^ above threshold, its enrichment factor in the Δ*PaClpP*/*HsClpP*^TRAP-TAG^ sample over the Δ*PaClpP*/*HsClpP*^WT-TAG^ sample is provided in parentheses.

^‡^If no human homologue was determinable, if possible a homologue from a fungal species was selected for reference.

^§^Protein whose prokaryotic homologue was identified as a substrate of *E*. *coli* CLPXP^20^,^21^.

^‖^Fe-S containing/binding protein. Listed are all proteins that were either exclusively or highly enriched co-purified with the catalytically inactive variant HsCLPP^TRAP-TAG^ and therefore classified as potential CLPXP substrates. For each protein, the *P*. *anserina* ID, average number of unique peptides identified by MS analyses across all biological replicates as well as Swiss-Prot ID, and gene and protein name of the human homologue (Supplementary Table 2) are listed. Categories (e.g. ‘Metabolism’) were assigned based on annotations from the Swiss-Prot database and the general literature.
