# Supplementary material for: Identification of potential mitochondrial CLPXP protease interactors and substrates suggests its central role in energy metabolism
Source: Sci Rep. 2015 Dec 17;5:18375. doi: 10.1038/srep18375 (PMC4683621; doi:10.1038/srep18375)
Supplement: Supplementary Information [file srep18375-s1.pdf]

Identification of potential mitochondrial CLPXP protease interactors and substrates suggest its central role in energy metabolism

Fabian Fischer, Julian D. Langer & Heinz D. Osiewacz

SUPPLEMENTARY INFORMATION

Supplementary Figures 1 to 2

Supplementary Tables 1 to 5

SUPPLEMENTARY FIGURES

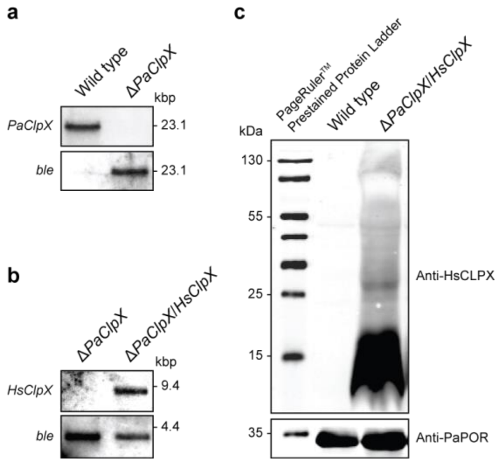

Supplementary Figure 1 | Human CLPX is not stable in *Podospora anserina* mitochondria.

a, Southern blot analysis of MunI-digested genomic DNA (gDNA) from a *P. anserina* wild-type and *PaClpX* deletion strain (Δ*PaClpX*). The *PaClpX*-specific probe detects the 17,715 bp fragment containing *PaClpX* only in wild-type gDNA. A 17,767 bp fragment containing the phleomycin resistance gene (*ble*) is detected by a *ble*-specific hybridization probe only in Δ*PaClpX* gDNA. **b**, Southern blot analysis of HindIII-digested gDNA with an *HsClpX*-specific hybridization probe verifying the *PaClpX* deletion strain containing an expression construct coding for human CLPX (Δ*PaClpX*/*HsClpX*). **c**, Western blot analysis of mitochondrial protein extracts from the indicated strains using an HsCLPX-specific antibody. Mature HsCLPX has a predicted mass of ~63 kDa. No corresponding signal is detectable in mitochondrial protein extracts from Δ*PaClpX*/*HsClpX*. PaPORIN was detected as a loading control.

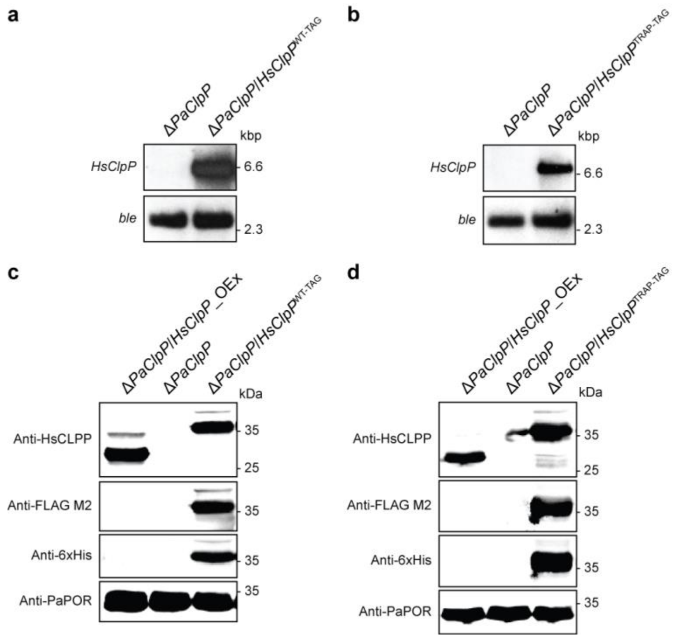

Supplementary Figure 2 | Verification of *PaClpP* deletion strains with human CLPP variants for use in a CLPP substrate-trapping assay.

a, Southern blot analysis of HindIII-digested gDNA with a *HsClpP*-specific hybridization probe verifying presence of an expression construct coding for C-terminally 3xFLAG-6xHis-tagged wild-type human CLPP in Δ*PaClpP*/*HsClpP*$^{\text{WT-TAG}}$. *ble*, phleomycin resistance gene. **b**, Verification of Δ*PaClpP*/*HsClpP*$^{\text{TRAP-TAG}}$ by Southern blot analysis as in a. **c**, Confirmation of the presence of affinity-tagged wild-type human CLPP in Δ*PaClpP*/*HsClpP*$^{\text{WT-TAG}}$ mitochondria by western blot analysis of mitochondrial protein extracts from the indicated strains using different antibodies. Mitochondrial protein extract from a strain expressing untagged wild-type human CLPP (Δ*PaClpP*/*HsClpP*_OEx) was used for comparison. PaPORIN was detected as a loading control. **d**, Confirmation of the presence of affinity-tagged inactive human CLPP in Δ*PaClpP*/*HsClpP*$^{\text{TRAP-TAG}}$ mitochondria by western blot analysis as in c.

SUPPLEMENTARY TABLES

Supplementary Table 1 | All proteins identified in the background control sample (Δ*PaClpP*) as well as in the samples containing HsCLPP$^{WT\text{-}TAG}$ or HsCLPP$^{TRAP\text{-}TAG}$.

P. anserina ID*	Protein	Avg. Number of Unique Peptides Δ*PaClpP*	Avg. Number of Unique Peptides HsCLPP$^{WT\text{-}TAG}$	Avg. Number of Unique Peptides HsCLPP$^{TRAP\text{-}TAG}$
Pa_6_2570	Putative Mitochondrial of precursor Heat shock protein SSC1	2.3	**27.0 (x 11.6)**	**28.3 (x 12.1)**
Pa_3_10800	Putative Sulfide:quinone oxidoreductase, mitochondrial precursor	14.0	16.0	18.7
Pa_1_8390	Putative mitochondrial ATP synthase subunit alpha precursor	13.0	15.7	12.7
Pa_6_5610	Putative Mitochondrial precursor of ATP synthase subunit beta	8.7	13.3	14.7
Pa_3_10150	Putative ADP,ATP carrier protein	9.0	10.0	9.3
Pa_3_4870	Putative NADH-ubiquinone oxidoreductase 78 kDa subunit mitochondrial precursor	2.7	3.7	**21.0 (x 7.9)**
Pa_3_10790	Putative Beta-lactamase hydrolase-like protein	1.3	**11.3 (x 8.5)**	**13.0 (x 9.8)**
Pa_2_9780	Putative outer mitochondrial membrane protein porin	7.7	9.0	8.3
Pa_1_5850	Putative mitochondrial isocitrate dehydrogenase [NAD] subunit 1 precursor	6.7	9.0	8.3
Pa_2_12010	Putative Elongation factor Tu, mitochondrial precursor	1.7	**8.7 (x 5.2)**	**10.0 (x 6.0)**
Pa_1_9290	Putative mitochondrial succinate dehydrogenase [ubiquinone] flavoprotein subunit precursor	5.7	7.7	6.7
Pa_6_8690	Putative protein of unknown function	7.3	8.7	3.7
Pa_6_5140	Putative Mitochondrial precursor of phosphate carrier protein	6.0	6.7	6.0
Pa_6_2730	Putative Isocitrate dehydrogenase [NAD] subunit 2	0.7	**7.3 (x 11.0)**	**6.3 (x 9.5)**
Pa_6_4730	Putative Mitochondrial precursor of Cytochrome b-c1 complex subunit 2	4.0	6.0	4.0
Pa_1_17000	Putative Woronin body major protein precursor HEX1	3.0	4.7	4.3
Pa_1_13390	Putative lipoamide acyltransferase component of branched-chain alpha-keto acid dehydrogenase complex	3.0	3.3	4.3
Pa_3_6450	Putative mitochondrial acetolactate synthase small subunit precursor	1.7	5.0	3.7
Pa_6_3510	Putative ATP-dependent RNA helicase mss116	2.0	4.0	4.3
Pa_1_17630	Putative mitochondrial ATP synthase subunit 4 precursor	2.0	4.0	3.0
Pa_2_11540	Putative Cytochrome c oxidase polypeptide VI, mitochondrial precursor	2.7	2.7	3.3
Pa_3_3200	Putative Acetolactate synthase catalytic subunit, mitochondrial precursor	3.0	2.0	3.7
Pa_3_8090	Putative mitochondrial ATP synthase subunit 5 precursor	2.7	3.0	2.7
Pa_1_7770	Putative protein of unknown function	2.3	3.0	2.3
Pa_7_570	Putative cytochrome c oxidase polypeptide IV	2.3	2.3	3.0
Pa_1_9450	Putative mitochondrial ATP synthase gamma chain precursor	2.0	2.7	2.3
Pa_2_1420	Putative mitochondrial LSU ribosomal protein L17 precursor	3.3	1.7	1.7
Pa_2_4980	Putative 3-hydroxybutyryl-CoA dehydrogenase	0.7	2.0	**3.3 (x 5.0)**
Pa_1_19590	Putative protein of unknown function	1.3	2.3	2.3
Pa_6_5480	Cytochrome c oxidase polypeptide 5 encoded by the cox5 gene	1.7	1.3	1.7
Pa_3_11310	Putative mitochondrial LSU ribosomal protein L24 precursor	2.0	2.7	0.0
Pa_1_6980	Putative prohibitin-2	1.3	1.7	1.3
Pa_3_9080	Putative mitochondrial LSU ribosomal protein L16 precursor	1.3	1.3	1.7
Pa_1_6520	Putative mitochondrial-processing peptidase subunit beta precursor	2.0	1.3	0.7
Pa_1_12550	Cytochrome c& encoded by the cyc1 gene	1.0	1.3	1.3
Pa_1_19720	Cytosolic translation elongation factor 1 subunit A (eEF1A) encoded by the AS4 gene	1.0	1.3	1.3
Pa_3_6870	Putative protein of unknown function	1.0	1.3	1.3
Pa_2_12340	Putative protein of unknown function	1.0	1.3	1.0
Pa_7_8595	Putative protein of unknown function	1.3	1.0	1.0
Pa_mito_cox2	Cytochrome c oxidase subunit 2	1.0	1.0	1.3
Pa_1_10210	Putative protein of unknown function	1.0	1.0	1.0
Pa_1_4450	Putative protein of unknown function	1.0	1.0	1.0
Pa_3_7390	Putative Kinesin-like protein	1.0	1.0	1.0
Pa_5_5380	Putative protein of unknown function	1.0	1.0	1.0
Pa_5_9300	Putative protein of unknown function	1.0	1.0	1.0
Pa_4_5910	Putative Stomatin-like protein	1.3	1.0	0.7
Pa_7_2170	Putative mitochondrial ATP synthase delta chain precursor	0.7	1.0	1.3
Pa_3_6770	Putative mitochondrial DNA replication protein YHM2	0.7	1.0	1.0
Pa_3_4100	Putative NADH-ubiquinone oxidoreductase 29.9 kDa subunit mitochondrial precursor	1.0	1.0	0.0
Pa_2_11200	Putative actin	1.0	0.0	0.7
Pa_4_7790	Putative Ubiquitin	0.7	0.7	0.0
Pa_5_6010	Putative NADH-ubiquinone oxidoreductase 40 kDa subunit	1.3	0.0	0.0
Pa_1_13340	mtHMG1 mitochondrial protein encoded by the mthmg1 gene	1.0	0.0	0.0

**P. anserina* IDs correspond to the '*P. anserina* Genome Project' database release version 6.32 (downloaded from http://podospora.igmors.u-psud.fr/)

For each protein, the *P. anserina* ID, protein name (based on the annotation from the '*P. anserina* Genome Project' database) and average numbers of unique peptides identified by MS analysis across all biological replicates of the respective sample are listed. Proteins that were highly enriched in the samples containing HsCLPP$^{WT\text{-}TAG}$ or HsCLPP$^{TRAP\text{-}TAG}$ over the background control sample (using spectral counting, enrichment factor > 3) are in bold (enrichment factor in parentheses).

Supplementary Table 2 | Human homologues of proteins co-purifying with HsCLPP^{WT-TAG} and HsCLPP$^{TRAP-TAG}$ in *P. anserina*.

P. anserina ID*	Avg. Number of Unique Peptides HsCLPP^{WT-TAG}	Avg. Number of Unique Peptides HsCLPP$^{TRAP-TAG}$	% Identity/% Positives *H. sapiens* homologue	E-Value	Swiss-Prot ID	Gene
**Chaperones and Protein Import**						
Pa_6_2570	27.0	28.3	66/81	0.0	P38646	HSPA9
Pa_6_5750	16.7	13.7	57/75	0.0	P10809	HSPD1
Pa_2_9700	14.3	15.3	29/47	2.8E-71	O94826	TOMM70A
Pa_2_10580	9.0	9.3	30/48	1.5E-25	O96008	TOMM40
Pa_6_1920	3.0	2.7	25/42	1.9E-7	Q15388	TOMM20
Pa_2_12760	1.7	1.3	57/76	3.8E-102	P35232	PHB
**Metabolism**						
Pa_1_22300	12.0	15.3	52/66	0.0	P23378	GLDC
Pa_3_10790	11.3	13.0	29/44	6.6E-19	O95571	ETHE1
Pa_5_5970	10.0	9.0	68/81	0.0	Q99798	ACO2
Pa_3_11290	8.7	9.3	63/77	1.1E-175	P00505	GOT2
Pa_6_1590	8.0	7.0	53/72	1.9E-136	P24752	ACAT1
Pa_3_6780	7.3	14.0	65/81	0.0	O75390	CS
Pa_6_2730	7.3	6.3	62/76	3.4E-137	P50213	IDH3A
Pa_3_2310	6.7	12.0	47/63	1.8E-121	P10515	DLAT
Pa_5_11920	5.7	5.0	35/52	1.7E-47	Q13825	AUH
Pa_2_1050	5.7	4.3	90/96	0.0	P78827†	ilv-2
Pa_6_10000	5.0	3.7	90/95	0.0	C7C436†	mcsA
Pa_1_13140	4.7	6.3	53/72	0.0	P31327	CPS1
Pa_3_7700	4.3	7.7	59/76	6.6E-172	Q9P2R7	SUCLA2
Pa_1_3450	4.0	6.0	32/49	1.7E-28	Q92506	HSD17B8
Pa_1_17280	3.3	5.0	58/73	0.0	Q12428†	PDH1
Pa_1_14630	3.0	6.3	25/45	4.1E-25	Q8N159	NAGS
Pa_4_7010	3.0	3.7	31/51	1.9E-30	Q16698	DECR1
Pa_3_10910	3.0	3.3	86/92	0.0	P15937†	acu-8
Pa_4_8600	3.0	3.0	44/61	4.4E-91	O15382	BCAT2
Pa_2_6200	2.3	5.0	38/60	4.2E-25	P23434	GCSH
Pa_6_8420	2.3	4.0	52/72	4.0E-137	P45954	ACADSB
Pa_2_430	2.3	3.3	71/86	0.0	Q9Y697	NFS1
Pa_3_2600	2.0	5.3	57/71	9.9E-117	P40926	MDH2
Pa_1_15690	2.0	3.7	54/65	0.0	Q10341†	cys2
Pa_2_4980	2.0	3.3	39/51	2.3E-54	Q16836	HADH
Pa_3_1420	2.0	2.0	34/51	2.4E-34	Q9UHQ9	CYB5R1
Pa_3_9430	1.7	3.0	31/48	1.8E-56	P04181	OAT
Pa_4_3040	1.7	1.7	63/75	0.0	P48735	IDH2
Pa_1_7660	1.7	1.3	58/72	0.0	Q02252	ALDH6A1
Pa_7_10210	1.3	3.0	54/72	3.7E-87	P30084	ECHS1
Pa_1_1980	1.3	1.7	55/70	0.0	P51649	ALDH5A1
Pa_4_660	1.3	1.3	57/74	0.0	P34897	SHMT2
**Electron Transport Chain and Respiration**						
Pa_1_14370	2.7	2.3	26/50	2.1E-11	O75947	ATP5H
Pa_4_7160	2.0	4.7	57/71	1.3E-75	O75489	NDUFS3
Pa_1_8620	2.0	1.7	72/86	0.0	O75306	NDUFS2
Pa_5_7500	1.3	1.7	42/65	5.5E-17	O14561	NDUFAB1
Pa_6_240	1.3	1.3	56/73	4.2E-74	P47985	UQCRFS1
**Other Pathways**						
Pa_2_12010	8.7	10.0	55/73	3.2E-160	P49411	TUFM
Pa_4_1130	2.3	2.3	nhd	-	-	Pa_4_1130
Pa_5_8240	2.0	2.7	47/59	2.1E-38	P30044	PRDX5
Pa_6_8740	2.0	2.3	41/55	2.9E-15	P10599	TXN

**P. anserina* IDs correspond to the '*P. anserina* Genome Project' database release version 6.32 (downloaded from http://podospora.igmors.u-psud.fr/)

†If no human homologue was determinable, a homologue from a fungal species was selected for reference

For each protein, the *P. anserina* ID, average number of unique peptides identified by MS analysis across all biological replicates of the respective sample, % of identical and positive amino acids compared with the human homologue's matched region, corresponding BLAST search E-value as well as Swiss-Prot ID and gene name of the human homologue are listed. Categories (e.g. 'Metabolism') were assigned based on annotations from the Swiss-Prot database and the general literature. nhd, no homologue determinable

Supplementary Table 3 | Human homologues of potential CLPXP substrates in *P. anserina*.

P. anserina ID*	Avg. Number of Unique Peptides	% Identity/% Positives *H. sapiens* homologue	E-Value	Swiss-Prot ID	Gene
**Chaperones**					
Pa_6_5510	7.3	38/54	5.0E-45	Q8NBU5	ATAD1
Pa_6_5590	3.7	36/52	2.5E-76	O76031	CLPX
**Metabolism**					
Pa_6_5560	15.7	46/64	0.0	Q02218	OGDH
Pa_6_1640	7.7	42/63	5.8E-93	P31327	CPS1
Pa_7_9520	7.3	32/46	1.3E-29	O00330	PDHX
Pa_5_5370	5.0	54/67	3.6E-126	P36957	DLST
Pa_7_10050	4.0	54/70	4.7E-123	P08559	PDHA1
Pa_1_13750	2.7	46/61	1.6E-94	P48728	AMT
Pa_5_5810	2.3	58/73	0.0	P09622	DLD
Pa_1_15800	2.0	63/80	1.0E-141	P11177	PDHB
Pa_1_20100	1.3	60/72	7E-144	P26440	IVD
Pa_3_9520	1.3	45/61	1.6E-88	P35914	HMGCL
**Electron Transport Chain and Respiration**					
Pa_3_4870	21.0	64/69	0.0	P28331	NDUFS1
Pa_4_7950	3.7	75/86	0.0	P49821	NDUFV1
Pa_5_9670	3.3	41/57	2.9E-91	Q5T2R2	PDSS1
**Other Pathways**					
Pa_3_11170	10.7	61/78	5.4E-142	O59778†	bio2
Pa_1_18430	2.7	30/45	3.0E-19	P22626	HNRNPA2B1
Pa_2_10680	2.0	52/74	5.9E-37	Q86SX6	GLRX5
Pa_1_6330	1.7	54/72	0.0	Q96RP9	GFM1
Pa_5_2590	1.7	46/69	2.7E-5	G2TRP3†	ymr31

**P. anserina* IDs correspond to the '*P. anserina* Genome Project' database release version 6.32 (downloaded from http://podospora.igmors.u-psud.fr/)

†If no human homologue was determinable, a homologue from a fungal species was selected for reference

For each protein, the *P. anserina* ID, average number of unique peptides identified by MS analysis across all biological replicates, % of identical and positive amino acids compared with the human homologue's matched region, corresponding BLAST search E-value as well as Swiss-Prot ID and gene name of the human homologue are listed. Categories (e.g. 'Metabolism') were assigned based on annotations from the Swiss-Prot database and the general literature.

Supplementary Table 4 | Full GO enrichment analysis (biological process) of human homologues of proteins co-purifying with HsCLPP$^{WT\text{-}TAG}$ and HsCLPP$^{TRAP\text{-}TAG}$ in *P. anserina*.

GO Term	Count	Expected	Fold Enrichment	*P*-value
carboxylic acid metabolic process	27	1.7	> 5	4.4E-24
small molecule metabolic process	34	4.4	> 5	6.1E-23
oxoacid metabolic process	27	1.9	> 5	1.3E-22
organic acid metabolic process	27	1.9	> 5	2.2E-22
oxidation-reduction process	25	2.0	> 5	4.1E-19
single-organism metabolic process	37	8.5	4.4	1.6E-17
cellular respiration	13	0.3	> 5	9.7E-14
organic acid catabolic process	13	0.4	> 5	4.8E-13
carboxylic acid catabolic process	13	0.4	> 5	4.8E-13
cellular amino acid metabolic process	15	0.7	> 5	7.7E-13
organonitrogen compound metabolic process	22	2.7	> 5	6.7E-12
energy derivation by oxidation of organic compounds	14	0.7	> 5	1.2E-11
small molecule catabolic process	13	0.5	> 5	1.7E-11
cellular metabolic process	40	16.6	2.4	3.8E-11
primary metabolic process	40	16.8	2.4	6.0E-11
**cellular amino acid catabolic process**	10	0.2	> 5	1.4E-10
**generation of precursor metabolites and energy**	14	0.8	> 5	1.6E-10
organic substance metabolic process	40	17.4	2.3	2.6E-10
alpha-amino acid metabolic process	12	0.5	> 5	3.6E-10
metabolic process	41	19.6	2.1	5.4E-10
**tricarboxylic acid cycle**	7	0.1	> 5	1.7E-09
citrate metabolic process	7	0.1	> 5	4.1E-09
tricarboxylic acid metabolic process	7	0.1	> 5	9.1E-09
aerobic respiration	7	0.1	> 5	1.1E-07
single-organism catabolic process	15	1.6	> 5	1.2E-07
small molecule biosynthetic process	11	0.8	> 5	1.8E-06
branched-chain amino acid catabolic process	5	0.0	> 5	3.5E-06
organonitrogen compound catabolic process	10	0.6	> 5	3.9E-06
branched-chain amino acid metabolic process	5	0.1	> 5	9.1E-06
single-organism process	41	24.9	1.7	9.9E-06
organic acid biosynthetic process	9	0.5	> 5	2.1E-05
carboxylic acid biosynthetic process	9	0.5	> 5	2.1E-05
single-organism cellular process	39	22.3	1.8	6.1E-05
cellular amino acid biosynthetic process	6	0.2	> 5	1.5E-04
cellular catabolic process	15	2.7	> 5	1.9E-04
alpha-amino acid catabolic process	6	0.2	> 5	2.1E-04
dicarboxylic acid metabolic process	6	0.2	> 5	2.2E-04
monocarboxylic acid metabolic process	10	1.0	> 5	2.7E-04
single-organism biosynthetic process	14	2.6	> 5	8.4E-04
**respiratory electron transport chain**	6	0.2	> 5	1.3E-03
electron transport chain	6	0.3	> 5	1.4E-03
glutamine family amino acid metabolic process	5	0.1	> 5	1.7E-03
catabolic process	15	3.3	4.5	2.7E-03
alpha-amino acid biosynthetic process	5	0.2	> 5	3.1E-03
organic substance catabolic process	14	2.9	4.9	3.1E-03
monocarboxylic acid catabolic process	5	0.2	> 5	5.4E-03
nucleobase-containing small molecule metabolic process	9	1.1	> 5	6.7E-03
serine family amino acid metabolic process	4	0.1	> 5	9.9E-03
nitrogen compound metabolic process	25	10.4	2.4	1.1E-02
**fatty acid metabolic process**	7	0.6	> 5	1.2E-02
cellular process	40	27.7	1.4	1.7E-02
organonitrogen compound biosynthetic process	9	1.2	> 5	1.7E-02
protein targeting to mitochondrion	4	0.1	> 5	3.7E-02
establishment of protein localization to mitochondrion	4	0.1	> 5	4.6E-02

Gene ontology (GO) enrichment analysis was performed online (http://amigo.geneontology.org/rte). Input were the Swiss-Prot IDs of all 41 human homologues of proteins co-purifying with HsCLPP$^{TRAP\text{-}TAG}$ and HsCLPP$^{WT\text{-}TAG}$ in *P. anserina* (see Supplementary Table 1). Only results with a *P*-value < 5.0E-02 are displayed. Selected representative or descriptive GO terms included in Fig. 3a are in bold.

Analysis Type: PANTHER Overrepresentation Test (release 20150430) | Annotation Version and Release Date: GO Ontology database; Released 2015-05-09 | Reference List: *Homo sapiens* (all genes in database) | Annotation Data Set: GO biological process complete | Bonferroni correction: true

Supplementary Table 5 | Full GO enrichment analysis (biological process) of human homologues of potential CLPXP substrates in *P. anserina*.

GO Term	Count	Expected	Fold Enrichment	*P*-value
**tricarboxylic acid cycle**	5	0.0	> 5	2.4E-07
**cellular respiration**	7	0.1	> 5	3.8E-07
citrate metabolic process	5	0.0	> 5	4.6E-07
alpha-amino acid catabolic process	6	0.1	> 5	6.3E-07
energy derivation by oxidation of organic compounds	8	0.3	> 5	6.8E-07
tricarboxylic acid metabolic process	5	0.0	> 5	8.2E-07
carboxylic acid metabolic process	10	0.7	> 5	1.4E-06
regulation of acetyl-CoA biosynthetic process from pyruvate	4	0.0	> 5	2.0E-06
**cellular amino acid catabolic process**	6	0.1	> 5	2.1E-06
small molecule metabolic process	13	1.8	> 5	2.6E-06
regulation of acyl-CoA biosynthetic process	4	0.0	> 5	2.7E-06
**generation of precursor metabolites and energy**	8	0.3	> 5	3.0E-06
alpha-amino acid metabolic process	7	0.2	> 5	4.5E-06
aerobic respiration	5	0.0	> 5	4.9E-06
oxoacid metabolic process	10	0.8	> 5	5.0E-06
organic acid metabolic process	10	0.8	> 5	6.0E-06
oxidation-reduction process	10	0.8	> 5	7.1E-06
regulation of sulfur metabolic process	4	0.0	> 5	1.2E-05
acyl-CoA metabolic process	5	0.1	> 5	1.3E-05
thioester metabolic process	5	0.1	> 5	1.3E-05
pyruvate metabolic process	5	0.1	> 5	2.2E-05
single-organism metabolic process	15	3.5	4.3	3.8E-05
cellular amino acid metabolic process	7	0.3	> 5	4.8E-05
organic acid catabolic process	6	0.2	> 5	6.0E-05
carboxylic acid catabolic process	6	0.2	> 5	6.0E-05
organonitrogen compound metabolic process	10	1.1	> 5	1.6E-04
coenzyme metabolic process	6	0.2	> 5	2.2E-04
regulation of coenzyme metabolic process	4	0.0	> 5	2.9E-04
regulation of cofactor metabolic process	4	0.0	> 5	2.9E-04
small molecule catabolic process	6	0.2	> 5	3.2E-04
lysine catabolic process	3	0.0	> 5	4.2E-04
lysine metabolic process	3	0.0	> 5	4.2E-04
single-organism catabolic process	8	0.7	> 5	6.7E-04
cofactor metabolic process	6	0.3	> 5	8.1E-04
organonitrogen compound catabolic process	6	0.3	> 5	1.1E-03
sulfur compound metabolic process	6	0.3	> 5	1.3E-03
aspartate family amino acid catabolic process	3	0.0	> 5	2.8E-03
purine ribonucleoside monophosphate metabolic process	5	0.2	> 5	3.2E-03
regulation of fatty acid metabolic process	4	0.1	> 5	3.3E-03
purine nucleoside monophosphate metabolic process	5	0.2	> 5	3.3E-03
acetyl-CoA metabolic process	3	0.0	> 5	3.9E-03
branched-chain amino acid catabolic process	3	0.0	> 5	3.9E-03
ribonucleoside monophosphate metabolic process	5	0.2	> 5	4.3E-03
organic substance catabolic process	9	1.2	> 5	4.6E-03
nucleoside monophosphate metabolic process	5	0.2	> 5	5.6E-03
branched-chain amino acid metabolic process	3	0.0	> 5	6.9E-03
**acetyl-CoA biosynthetic process from pyruvate**	2	0.0	> 5	9.5E-03
catabolic process	9	1.4	> 5	1.5E-02
purine ribonucleoside metabolic process	5	0.2	> 5	1.6E-02
purine nucleoside metabolic process	5	0.2	> 5	1.7E-02
nucleobase-containing small molecule metabolic process	6	0.4	> 5	2.2E-02
ribonucleoside metabolic process	5	0.2	> 5	2.4E-02
purine ribonucleotide metabolic process	5	0.3	> 5	2.7E-02
metabolic process	17	8.1	2.1	2.7E-02
ribonucleotide metabolic process	5	0.3	> 5	3.4E-02
nucleoside metabolic process	5	0.3	> 5	3.5E-02
purine nucleotide metabolic process	5	0.3	> 5	3.7E-02
cellular catabolic process	8	1.1	> 5	3.9E-02
ribose phosphate metabolic process	5	0.3	> 5	4.0E-02
glycosyl compound metabolic process	5	0.3	> 5	4.6E-02
phosphorus metabolic process	9	1.6	> 5	4.7E-02

Gene ontology (GO) enrichment analysis was performed online (http://amigo.geneontology.org/rte). Input were the Swiss-Prot IDs of all 18 human homologues of potential CLPXP candidate substrates identified in *P. anserina* (see Supplementary Table 2). Only results with a *P*-value < 5.0E-02 are displayed. Selected representative or descriptive GO terms included in Fig. 3b are in bold.

Analysis Type: PANTHER Overrepresentation Test (release 20150430) | Annotation Version and Release Date: GO Ontology database; Released 2015-05-09 | Reference List: *Homo sapiens* (all genes in database) | Annotation Data Set: GO biological process complete | Bonferroni correction: true
